# Horizontal Review on Video Surveillance for Smart Cities: Edge Devices, Applications, Datasets, and Future Trends

**DOI:** 10.3390/s21093222

**Published:** 2021-05-06

**Authors:** Mostafa Ahmed Ezzat, Mohamed A. Abd El Ghany, Sultan Almotairi, Mohammed A.-M. Salem

**Affiliations:** 1 Faculty of Media Engineering and Technology, German University in Cairo, Cairo 11511, Egypt or mostafa.kamal-ezzat@student.guc.edu.eg (M.A.E.); or salem@cis.asu.edu.eg (M.A.-M.S.); 2 Electronics Department, German University in Cairo, Cairo 11511, Egypt; mohamed.abdel-ghany@guc.edu.eg; 3 Integrated Electronic Systems Lab, TU Darmstadt, 64283 Darmstadt, Germany; 4 Department of Natural and Applied Sciences, Faculty of Community College, Majmaah University, Majmaah 11952, Saudi Arabia; 5 Faculty of Computer and Information Sciences, Ain Shams University, Cairo 11566, Egypt

**Keywords:** smart city, IOT, computer vision, surveillance

## Abstract

The automation strategy of today’s smart cities relies on large IoT (internet of Things) systems that collect big data analytics to gain insights. Although there have been recent reviews in this field, there is a remarkable gap that addresses four sides of the problem. Namely, the application of video surveillance in smart cities, algorithms, datasets, and embedded systems. In this paper, we discuss the latest datasets used, the algorithms used, and the recent advances in embedded systems to form edge vision computing are introduced. Moreover, future trends and challenges are addressed.

## 1. Introduction

In the past few years, cities around the world have been starting to develop modern smart city infrastructure that mostly cannot be developed without the use of the latest technology. This technology can lead to a change in a city’s organizational framework and provide a data-driven perspective on management. Digital Transformation has become a global demand for all people living in cities. This enhances the lifestyle of citizens living in the country.

Smart Cities provide a better living standard and make people feel safer with 24/7 security. This comes with also taking into consideration the privacy of individuals living in the city. As people use the applications, their data are processed. These data are analyzed by developers. For this reason, some laws must be implemented to govern the use of the data. Monitoring these cities is also done by the use of Digital Twins, which connect and monitor the city and run simulations based on the data.

Video Surveillance is one of the main and crucial building blocks of smart cities. Smart surveillance is a new initiative that sets a higher ceiling for the future of smart cities. Video Surveillance offers people more tools and applications to monitor with fewer human mistakes that improves the city’s stability. There is more than one component that completes the cycle of Smart Surveillance. This will be discussed later in the paper.

Through advances in Artificial Intelligence (AI) and Computer Vision came the use of Edge Computing and Embedded components, which improved the integration of Smart Surveillance. This development created a strong relationship between IoT and edge computing. AI systems are becoming more and more advanced, leading to lots of enhancements in applications of new technologies.

Our paper expands all the previous subjects together with the deep organization of all fields. We comprehensively address leading smart city ecosystems created by edge computing, edge computing development, and open research challenges, unlike the current surveys. Our main contribution is that we added the latest edge computing technologies in the field of smart cities. We added the recent Video Surveillance advancements to every aspect and we reviewed the literature on recent fields in computer vision applications such as People Counting, Age and Gender Estimation, Action Recognition, Fire and Smoke Detection and Vehicle Detection. Another important contribution is that we gave a complete overview of the recently used datasets by giving a brief description of every dataset.

This paper is organized as follows—Section 2 of this paper briefly describes all the background of smart cities and video surveillance with an overview of all the recent review papers. Section 3 is a summary of the background of Embedded Systems and different architectures in today’s Computer Vision with a deep dive into the different algorithms used. Section 4 discusses the deep insights over Computer Vision applications such as people counting, crowd estimation, action recognition and abnormal action recognition. This section also states a comparison of methods used in the recent studies of Computer Vision applications in the applications mentioned above. Section 5 includes future trends in surveillance in smart cities. Section 6 states the conclusion of all the recent topics.

## 2. Background

In this section we’ll be summarizing the main related works to the topic we have. The related work consists of the different terminologies and definitions of smart cities. How smart cities are enhancing the security of people living in. Moreover, this section describes the different video surveillance components that are used. Also, this section describes edge computing and how embedded systems are becoming a big part of the main building blocks of edge computing. Then, discussing the relationship between IoT and edge computing. Lastly, discussing the existing literature review in the scope of our study by giving an overview on the different subjects such as Smart City, Surveillance, IoT, Artificial Intelligence and Edge Computing.

### 2.1. Smart Cities

There have been different definitions of smart cities. The term is popularly known but used with different names around the world and in various circumstances. The meaning of smart in the word “smart city” comes from the integration of many fields in computing technology. The term stands for the integration of cloud, network and end-user devices. Washburn et al. [1] stated that cities are getting “smarter,” as cities, enterprises and neighborhoods are increasingly dependent on technology to address accelerated urbanization challenges.

Giffinger et al. [2] described a Smart City as a well-performing community based on the ‘wise’ mix of self-decisive, autonomous and active resident endowments and activities. Hall et al. [3] stated that the vision of “Smart Cities” is the urban core of the future, rendered clean, stable, green and effective since all infrastructure whether for electricity, water and transportation is built and connected to a complete system of databases.

A smart city strives to truly realize intelligence in different social facilities, such as the lives of citizens, community services, defense and education. Smart cities use the latest technology such as the Internet of Things (IoT) sensors to collect data around the city. Decision-makers then use the knowledge obtained from this data to efficiently control properties, capital and services in the metropolitan environment. The main pillar behind all these technologies is using IoT sensors to gather data, which is the operational model of a smart city.

By 2025, more than 30 billion IoT users will link to the network, according to Gartner’s estimation. Urban projects that label themselves smart cities are increasing worldwide. Egypt and many other countries in the Middle East and North Africa region are working hard to convert their capital cities into smart cities. Perhaps the project of the “New Administrative Capital” in the eastern desert in Egypt is currently leading and is one of its kind in the region. There is also a mega project being implemented between KSA, Egypt and Jordan, called the Neom smart city.

For example, to improve security, Tokyo will use the latest technology of driverless cabs that are expected to take both athletes and people coming to watch the Olympics from one place to another. All of this is done without any human intervention. Seoul introduced the sharing parking service, which simply implements IOT sensors all over the parking space, giving citizens notifications of free places in public parking areas.

Thus, the opportunity to build new facilities with a better view of people’s behavior and the practical utilization of existing infrastructures is accomplished by obtaining this data. The strong advantages of the technology of smart cities have inspired many countries to put their efforts into financing big projects in the sector of building and developing smart cities projects.

Smart cities manage their operations by using all available information and communications technologies in the urban environment, thus improving the quality of life and driving economic development. With data being taken from users and devices, a crucial risk arises to the privacy of people. The General Data Protection Regulation (GDPR) has positioned some restrictions to enact complete fairness between both the end-users and the developers. The main steps in such an action are the known privacy risks, governed policies and the provisions.

Smart cities include many advances in the field of information and communications. This makes them vulnerable to threats such as cyber-attacks. Such attacks are Brute-force attacks, Credential Stuffing, Phishing, Denial of service (DOS) and Malware attacks. To solve these problems, public data access is restricted. To use it, you must first register and receive clearance, then track and control access and usage, and onduct penetration testing on all city services and networks daily.

Digital twins are being redefined as digital replications of living and nonliving entities that enable data to be seamlessly transferred between the physical and virtual worlds. They were created to enhance manufacturing processes.

Future smart cities will focus on developing systems that can meet the computational demands of expanded digitized data and related advanced software in fields like health and wellness, protection and safety, transportation and energy, mobility and communications, and transportation and energy.

### 2.2. Video Surveillance

Safety has always been a concern for cities in the past 10–15 years. With the increase in research in many fields in technology, it has become easier to enhance the safety and security of pedestrians living in cities including smart traffic systems and routes and smart safety systems for surveillance.

**Figure 1:**
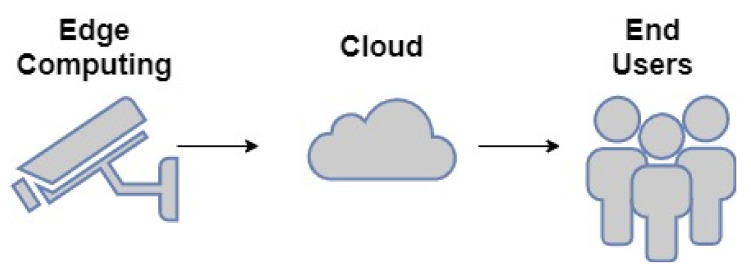
Complete System Architecture.

Figure 1 shows video surveillance in smart cities [4]. To the left of the figure are the edge computing components that are responsible for capturing the data and passing it to the Smart City inhabitant management (Cloud), which takes the data and tries to get information. It is then passed to the end-user completely analyzed. In Figure 1 starting with the edge computing, the front-end camera acts as the first action that captures in real-time, both with the video and feature extraction.

Feature extraction is the process of taking what is most important from a video, whether an action or a certain object predefined to be extracted. Then comes the step where video and features are encoded. Videos are compressed by this process to store them more efficiently. Data are sent to the cloud by a network. In the cloud interface, video decoding and feature decoding take place; this process could be saved as the method previously processed from the edge computing, and then kept in service for storing. The end-users simply use multiple applications once the data are stored on the cloud, such as people counting, crowd estimation, action recognition, age and gender estimation, vehicle estimation and vehicle counting and tracking.

#### 2.2.1. Edge Computing Component

Edge computing, needless to add, has become a major building block in the IoT world. Edge data mining lowers bandwidth usage and decreases delays in communication, helping end-users to make quicker decisions in sensitive circumstances. Edge computing mainly relates to the collection of data in the camera itself in video surveillance, which has multiple advantages.

Among end-user organizations, edge computing has become a more common and widespread term. Although data flies from endpoints to the cloud in 150 to 200 milliseconds, it takes only 10 milliseconds from endpoints to edges. In multiple verticals, this facilitates more efficient detection and reaction.

Due to the recent advancements in technology, cameras are starting to process the data themselves. Recent cameras include some IP cameras that can do deep-learning analytics and machine learning.

**Figure 2:**
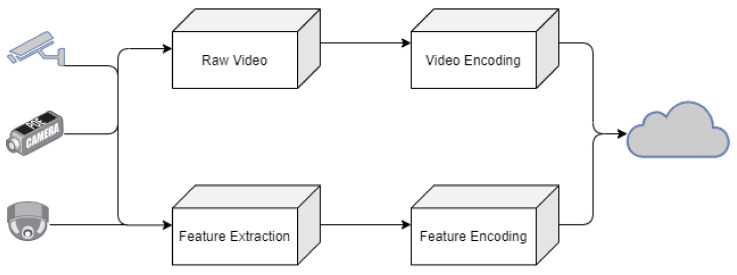
Video Management Components.

As seen in Figure 2, the whole system works in helping day-to-day surveillance. More and more IP camera manufacturers are demanding the new technology of machine learning to be integrated with the IP cameras. So, Bosch, one of the leading companies, started having it as its first commercial product, providing the chance to do video analytics with the system. FLIR cameras also started to be able to detect the surroundings using machine learning and also became capable of video analytics.

#### 2.2.2. Smart City Inhabitant Management (Cloud)

**Figure 3:**
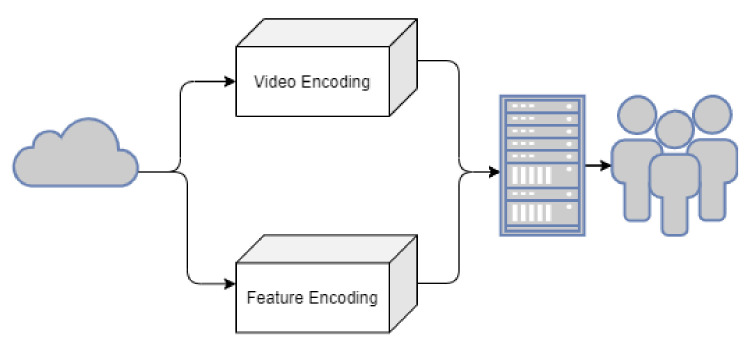
Cloud Services.

As seen in Figure 3, the cloud component’s main objective is linking the edge component, taking the distribution of both encoding the video and features. The Video and Feature Encoding are both stored to be easily accessed by the end-users.

#### 2.2.3. End Users

**Figure 4:**
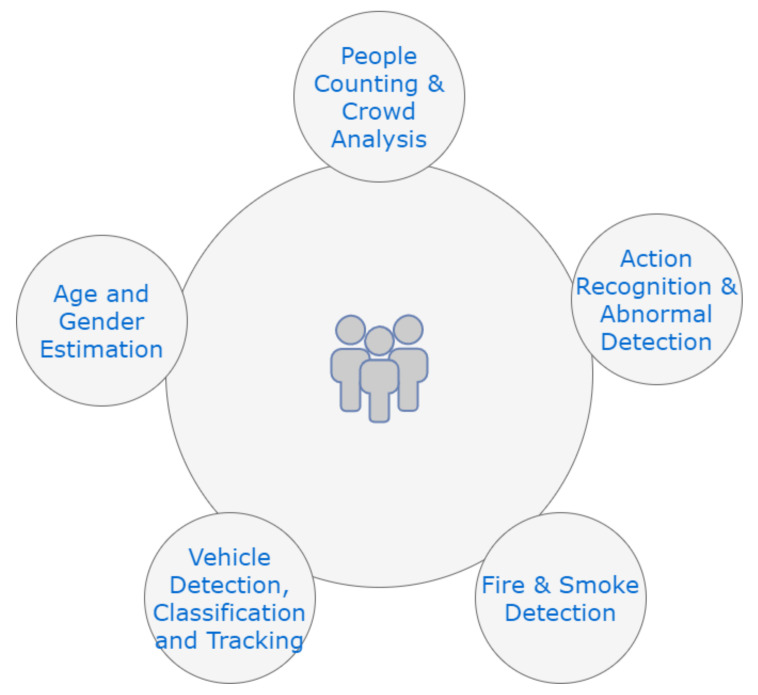
End Users Applications.

As seen in Figure 4, end-users have the luxury of using the data retrieved from the cloud for many options. These options can be People Counting, Age and Gender Estimation, Action Recognition, Fire and Smoke Detection, and Vehicle Detection.

### 2.3. Edge Computing Video Surveillance

Embedded Systems have helped in advancing the combination of computer hardware and software. With more advancements in the area of Embedded Systems, Machine Learning engineers are now integrating large systems to undertake a specific job. With more complex algorithms in Machine Learning comes higher computing power. Embedded Systems have been an important factor that should be discussed when coming to integrate large complex Machine Learning operations.

Systems operated by embedded sensors are trained to be fed with real-time data to detect possible issues. A neural network, however, applies multiple algorithms to resolve the problem at hand as an alternative approach. To study AI and Machine Learning methods that are only hypotheses and principles, embedded systems dealing with the science of combining hardware and the associated applications on a nanoscale can be used. For example, a robot is an embedded device (with chips, sensors, etc.) that runs software that can simulate AI and machine learning tasks, such as locating routes, recognizing faces, aggregating environmental data and submitting them to information representation servers.

Ren et al. [5] proposed an object detection architecture based on edge computing for real-time surveillance applications that achieved distributed and efficient object detection via wireless communications. It presents the proposed architecture as well as its possible advantages, as well as the problems that could arise during implementation.

From videos taken from cameras placed on the wall to cover a target area, their method could detect and recognize a human target. The proposed method entails detecting and monitoring any potentially dangerous targets. After that, the decision-making process determines whether or not the individual is a threat [6].

Edge computing is used to collect and process vast amounts of data from wireless sensors on a large scale. The IPFS storage service is used to store large amounts of video data, while CNN technology is used for real-time monitoring [7].

Acharya et al. [8] introduced the recent developments in neural network architectures that have made it possible to train algorithms on large datasets without having to manually tune them. For instance, they used a Faster R-CNN neural network architecture to monitor artifacts in CCTV footage. They demonstrated that using heterogeneous data and augmenting it with motion blur during the training process will improve the detector’s efficiency.

### 2.4. IOT and Edge-Computing Surveillance

With increased technology worldwide, scientists are trying to make all technologies work together and to integrate the latest technologies. For this purpose, scientists nowadays connected both the IoT and Edge-Computing Surveillance. Fu et al. [9] proposed that the overload events of relay nodes, base stations and communication links drive the cascading phase of IoTs. A load-oriented base station architecture scheme is presented to help IoTs increase network survivability.

Fu et al. [10] introduced the multi-sink WSNs and proposed a realistic cascading model. Two load metrics are proposed in this model to describe the load distributions of sensor nodes and wireless links, and the network’s cascading mechanism is jointly promoted by node and connection overload events, which can better represent the cascading characteristics of multi-sink WSNs in real-world scenarios. Also, they concentrate on the network’s cascading robustness in the face of node and connection attacks.

Both node and connection capacity has important thresholds that can decide if capacity expansion is beneficial. When it comes to network load distribution, there is a crucial level that can decide whether or not cascading failures occur. Node attacks are more likely than link attacks to cause cascading failures. Increased node capacity may help mitigate the network’s harm from cascading failures.

### 2.5. Surveillance Technologies in Smart Cities Digest

There have been many advancements, according to the literature review, in the area of Smart Cities, Surveillance, the Internet of Things, Artificial Intelligence and Edge Computing. Table 1 shows the latest papers with the area of specification. Zhaohua et al. [11] & Dlodlo et al. [12] introduced in their papers the current deployment of IoT in smart cities in different fields. The current development of federated learning from the Internet of Things, transportation, communications, banking, medical and other fields. They experimented with the development of urban areas that have advanced their infrastructure and how this increased in return the quality of city living. Those papers also shed a light on smart health, ambient-assisted living, crime prevention, and community safety.

Gharaibeh et al. [13] discussed data processing strategies that are used to ensure smart IoT system data reuse-ability, granularity, interoperability, and accuracy. Besides, in smart cities, the author’s defined the strategies used for protection and privacy. Recent advancements in surveillance when connected to edge computing are cited in [14,15,16,17]. Hu et al. [15] provide a detailed overview of three typical edge computing technologies, which is presented in this article, namely mobile edge computing, cloudlets, and fog computing. In brief, it outlines and contrasts the standardization efforts, concepts, designs, and implementations of these three technologies. Roman et al. [14] provided an overview of fog computing model design, core technology, implementations and problems, and open issues are outlined.

The key purpose of this paper is to examine the security risks, challenges, and processes inherent in all edge paradigms systematically while emphasizing possible synergies and cooperation locations [17].

Yu et al. [16] conducted a detailed review, exploring how edge computing enhances IoT network efficiency. Yu et al. [16] categorized edge computing into various architecture-based classes and examined their efficiency by analyzing network latency, occupancy of bandwidth, energy usage, and overhead. In [13,18,22,23,24,25] authors summarized the recent advancements in smart cities with reference to surveillance, artificial intelligence, and IoT. Gharaibeh et al. [13] identified the data protection and privacy strategies used. Addressing the network and computation technologies that allow smart cities, [22] provided conclusions from an overview of multiple use cases of big data in cities around the world and presented four initiatives by the authors with government agencies to build smart cities. This paper explicitly classifies urban data usage cases for smart cities in translating data into information. Chen et al. [23] presented an analysis from two viewpoints on the new studies on the integration of deep learning and smart cities; while the technique-oriented analysis pays attention to the common and expanded deep learning models, the technology-oriented review highlights the smart cities’ representative implementation domains.

## 3. Embedded Systems in Computer Vision

The Embedded system has been introduced as a powerful tool when considering integrating the software with hardware. Embedded Systems have been enormously integrated into many applications. Recent studies aim to move Embedded Systems into the next level of Machine Learning, Artificial Intelligence, and Deep Learning. Many of these advancements come from enhancing old systems with intelligence.

An embedded system is a hardware and software system based on microprocessors or microcontrollers designed for performing dedicated functions within a larger mechanical or electrical system [26]. Embedded Systems have been a building block in the evolution of smart cities. Many researchers are currently taking steps toward enhancing and building a mixture between the new Machine Learning approaches and the success in the world of Embedded Systems. This approach can be seen in smart and autonomous cars. Autonomous cars have cameras in the front of the car which send messages to stop or speed or keep distance between the car in front. The car also has sensors that send to the cloud many data to obtain feedback on the performance.

The embedded Computer Vision system has also been used for detecting anomalies, such as abnormal activities in the street, and for sending millions of data through sensors installed everywhere. With facial recognition, it became easier to detect the faces when searching for certain people. Home Automation has also been used in the advancements of Embedded Systems. Connecting all devices in the house to a system that can be easily controlled even from a distance [27].

Table 2 depicts the recent advancements in the Embedded Systems used in the field of Machine Learning of COB (computer-on-board). Table 2 shows the products dominating the process of Machine Learning from widely known hardware and software companies. NVIDIA is one of the leading companies when it comes to GPUs. Google has been a leading company in the industry of Machine Learning and Artificial Intelligence. Raspberry PI is one of the leading small single-board computers developed around the globe. Toradex is one of the leading companies in highly miniaturized embedded computing solutions. The Inforce 6601 Micro SoM is based on the latest embedded processor. Table 2 shows the advantages and disadvantages of each product recently used in COM (computer-on-model).

An integrable circuit with on-board hardware components, such as the GPU, CPU, and WLAN Card, is known as Computer-On-Board Architecture. It makes use of the bus mechanism, which allows hardware components like RAM, ROM, GPU, and CPU to connect. The architecture of the DSP: Harvard Architecture is used by a DSP processor, which is a special form of microprocessor that is designed for digital signal processing needs. It processes digital signals with the aid of digital-to-analog and analog-to-digital converters. It is mostly used for signal measurement, filtering, compression, and decompression. It can successfully convert a signal from one domain to another.

ALU, MAC, and shifters are examples of computer-on-board architecture that have more advanced computational optimized units for their purpose applications. Pipelining is now possible. This makes it a good board for checking processor architectures rather than using them in real-world processing. Many output peripherals on the FPGA board will imitate most boards, including LCDs, RAM, ROM, ADC/DAC, LEDs, pushbuttons, DIPs, and connectivity ports [32].

ASIC refers to any integrated circuit that is not reprogrammable. It has a high level of energy efficiency. The CPU in phones, for example, is called an ASIC, and although programmers will program these processors for various reasons, this programming would be limited to the instruction set that the processor was permanently programmed for. If, for example, high-level programming must be compiled to low-level programming using instruction from a pre-designed instruction set, the program would not run [33].

Table 3 consists of the different techniques and approaches that the Machine Learning use of Embedded Systems technologies. Table 3 shows that the ASIC has the advantage of power over FPGA but they are hard to manufacture as they are high in price so they are more used when it comes to high production levels. FPGA is easily manufactured and easy to test when it comes to testing the architectures of the processors. GPU is used to enhance the use of motions and display of images. GPUs are used frequently over the CPU when it comes to higher computational levels. Different algorithms are tested on different hardware types such as FPGA, ASIC, CPU, and GPU. Algorithms shown in Table 3 comes different types from simple Machine Learning algorithms ( PCA/ SVM/ Marakov Chain) to advanced studies of Deep Learning ( MLP/DNN/YOLO/RCNN/ANN/TABLA). Table 3 shows the frequency, latency and power of each algorithm alongside its hardware type.

Table 3 represents the different structure of the MLP topologies. As shown as the topology in the MLP complexity increase the frequency used increases with this also the latency decrease in time with also the power varies with different amounts. When it comes to Principal Component Analysis (PCA) there comes two types Decision tree and K-Nearest Neighbors (KNN) Algorithm. As seen the embedded system used is Zynq SoC ZC702 as it goes through complexity used the latency increases. Deep neural network (DNN) has experimented with four different types of ASICs (ASIC ACCELERATORS Systolic-ASIC ACCELERATORS Eyeriss-ASIC ACCELERATORS MAERI-ASIC ACCELERATORS MERIT-z). ASIC ACCELERATORS MERIT-z had the highest frequency with 400 MHz. The highest power is ASIC ACCELERATORS MERIT-z and the least power is ASIC ACCELERATORS Systolic.

Table 3 shows the multilayer perceptron (MLP) which is a feedforward artificial neural network which is a kind of feedforward artificial neural network (ANN).

There are at least three layers of nodes in an MLP—an input layer, a hidden layer, and an output layer. Each node, except for the input nodes, is a neuron with a nonlinear activation function. Backpropagation is a supervised learning method used by MLP for teaching. MLP is distinct from a linear perceptron by its many layers and non-linear activation. It can tell the difference between data that isn’t linearly separable.

PCA is a mathematical technique for defining underlying linear structures in a data set such that it can be represented in terms of other data sets with a significantly lower dimension with minimal knowledge loss. DT stands for Decision Trees. One of the statistical simulation methods used in analytics, data processing, and machine learning is decision tree learning. It goes from assumptions about an object (represented in the branches) to predictions about the item’s target value using a decision tree (as a predictive model) (represented in the leaves).

A Principal Component Analysis (PCA) is used to minimize redundancy information and data dimensionality using a K-Nearest Neighbor (KNN) regression. In a PCA-KNN model, a sliding window generates the historical data set as input, which is then converted by PCA into principal components with rich details, and then fed into KNN for prediction.

Deep neural networks (DNN) are a dominant class of machine learning algorithms that employ layers of neural networks stacked along with the depth and width of smaller architectures. In recent years, deep networks have shown discriminative and representation learning capacities across a diverse variety of applications. Deep learning’s horizons are being broadened by researchers in machine learning, looking for potential applications in various fields.

YOLO was created to aid the performance of slower two-stage object detectors like Faster R-CNN. R-CNNs are reliable, but they are slow even when they are running on a GPU. Single-stage detectors, such as YOLO, on the other hand, are very fast and can achieve super real-time efficiency on a GPU. Tiny-YOLO is a smaller clone of its big brothers, which means it is, therefore, less reliable.

For two-group classification problems, a support vector machine (SVM) is a supervised machine learning model that uses classification algorithms. SVM models will categorize new text after being given sets of named training data for each type.

Markov chains used in some systems-based on GPUs as shown in Table 3. A Markov chain is a stochastic model that represents a set of potential events where the probability of each occurrence is solely determined by the condition achieved in the previous event.

## 4. Computer Vision Applications

Computer Vision is an area of Artificial Intelligence that enables the computer to recognize its environment. Using digital images from cameras and photos, computers can identify and distinguish objects. The first phase in protecting indoor and outdoor facilities and adding additional protection has been done by video monitoring. Adding to the normal system a new impact on the advancement of Machine Learning. In this section, there will be an introduction to the techniques and approaches in the new field of Computer Vision. One of the main contributions of the research in Computer Vision applications in video surveillance is People Counting [43] and Crowd Analysis, Action Recognition [44], Vehicle Detection, Classification and Tracking, Fire and Smoke Detection [45] and Gender Estimation [46]. In this section, we tried to shed a light on recent computer vision applications with deep details on different data sets widely used with explanations on every dataset’s specifications.

Many approaches were used throughout the years in the field of Computer Vision in enhancing the solutions when it comes to security such fields are in people counting, crowd estimation, action recognition, abnormal action recognition, age, and gender estimation, vehicle estimation, and vehicle counting and tracking. Those advancements have always been into consideration. Over the years the Machine Learning algorithms are trying on pushing the limits. Researchers started from the beginning by using simple conventional techniques such as foreground-background subs traction. With the advancing of computational power, it became easier and easier in enhancing the algorithms. Then came Machine Learning which added new techniques in detecting and higher accuracy. More and more enhancements came by. With the introduction of Deep Learning techniques like Neural Networks with much deeper enhancements and advancements came new ways of enhancing.

### 4.1. People Counting and Crowd Analysis

Many video processing algorithms and Computer Vision-based approaches for monitoring and people counting are developed in recent years to deal with various applications. Researchers are trying to build models to identify the context and monitor the motion.

Keem et al. [43] proposed a method of detecting and monitoring moving persons by using bounding boxes to enclose each individual. In the supervised sector, an approximate convex hull is obtained for each person to provide more precise tracking data. There are different ways to count people in Computer Vision. The most used today practically are Detection Based, Cluster-Based, Feature-Regression and Neural Network. Starting with the oldest of the algorithms above is the Detection Based. Detection Based is trained by labeled data with a training set which consists of a body picture of people. The classifier then takes the pictures and trains over them. The accuracy for the proposed method is 96%.

The classifier tries to get patterns from the training dataset. Examples of the classifier used are the Radial basis function kernel using Support Vector Machine and Random Forests. The system works well when it comes to detecting faces but less well when coming to people detection, as the dataset given had different shapes and sizes of people which makes it difficult for the classifier. It also suffers from occlusion in crowded scenes, and scene distortion is unavoidable. For surveillance applications, it works much worse, where pictures of very low-resolution [47]. The second algorithm uses an unsupervised cluster Based algorithm in which it assumes everything is relatively unique and constant. Examples of Cluster-Based algorithms are Bayesian Clustering and KLT Tracker [48].

The feature Regression algorithm requires identifying the region of interest perspective map, then extract low-level image features in front of the region, such as foreground pixels or image edges.The most recent algorithm used is Neural Networks which is a system build from end-to-end to use both regression and classification. These images that enter the model are fine-tuned to get most of the features to make it better in finding the patterns in the photos which relatively gives a better amount of counting people. The main advantage of such an algorithm is that it is better used in surveillance and in getting the highest accuracy in counting the number of people whether it is in a low-quality or high quality video. Even the place if it was crowded much this can be used in counting very quickly with good accuracy better than the algorithms that are given above [49,50].

Crowd Estimation has been a great challenge to scientists. The main challenge is that crowds can be classified into different groups. These groups are hard to classify as, for example, it can be difficult to detect which ethnic groups a biracial person belongs to. Another challenge is also that skin color can change with the seasons.

There have been many approaches over the years. Wu et al. [51] introduced crowd density estimation using texture analysis and learning with an accuracy of 69.9%. An et al. [52] implemented face recognition using kernel ridge regression with an accuracy of 91%. Chan et al. [53] introduced privacy-preserving crowd monitoring of counting people without people models or tracking. Lemptisky et al. [54] produced object recognition from local scale-invariant features with an accuracy of 82%. Chen et al. [55] proposed feature mining for localized crowd counting with an accuracy of 74.5%. The conventional approach has been the basic block in analysis [56]. Table 4 summarizes the most-used datasets in the field of people counting and analysis.  UCSD is a stationary camera mounted at an elevation overlooking pedestrian walkways that was used to capture the UCSD Anomaly Detection Dataset. The density of people in the walkways ranged from minimal to very crowded. The video includes only pedestrians in its default environment. Abnormal incidents can be triggered by one of two things—the passage of non-pedestrian individuals across walkways atypical pedestrian activity trends.  UCFCC50 images with highly dense crowds can be found in this data collection. The photographs were also taken from the FLICKR website.

Table 5 shows the different methods used for people counting and crowd analysis. There are different methods when it comes to a conventional method like spatial features that are sensitive to background and illumination. Then comes the Machine Learning technique, which can be seen using CNN. With deeper and more sophisticated advancements of research and design, Deep Learning introduced new techniques such as the Gaussian Activation Map (GAM) and RetailNet which can detect and count people in crowd areas easily. This table presents methods with their advantages and disadvantages with references to papers for every method.

### 4.2. Age and Gender Estimation

Age estimation has always been a hot topic for discussion. Nowadays with the introduction of Machine Learning, things became easier. The earliest approaches to the human face used scale and proportions. Those approaches are limited to young people and to the nature of the human head, which changes considerably in adulthood. In this section, there will be a discussion on the different approaches to age estimation.

Approximate age was used in the past to manually extract facial information, but now CNN methods [46] preferred results by CNN acting directly on datasets on age. Face-age datasets are broken down into datasets with biological ages and age sets, and these age datasets will be used for various methods with age measurement. Zhang et al. [46] introduced a proposal for a prediction scheme for age and gender that integrates a residual multi-level network of systems that are used to produce findings for the public benefit benchmark. Gao et al. [63] suggested a wide set of Learning Process (DL).

Demirkus et al. [64] provided the first of its kind of research into the recognition of faces in natural environments from ungoverned video sequences. To describe temporal dependencies, a Markov model is used, and classification includes evaluating the maximum posterior. According to the algorithm in [65], SIFT features obtained from training images are clustered to learn the most effective characteristics differentiating between females and males.

In his paper, Nguyen et al. [66] proposed a new gender recognition approach for identifying males and females in surveillance system observation scenes based on feature extraction via CNN. Since the CNN model is equipped with gender knowledge using a vast number of human body images, they combined the image characteristics derived (by the CNN method) from visible-light and thermal images and perform noise and feature dimension reduction by main component analysis (PCA).

Arigbabu et al. [67] presented a way to deal with gender recognition. Their supposition that is principally founded on the actuality that a single image is accessible for every person in the database, their methodology includes extracting face shape description by consolidating Laplacian separated pictures with Pyramid Histogram of Gradient (PHOG) shape descriptor, introduced by [68] to help gender recognition. Table 6 has the frequently used datasets in the field of age and gender estimation. SCFace is a series of static human face pictures. Five video surveillance cameras of differing quality were used to capture photographs of an unregulated indoor setting. There are 4160 static photographs (in the visible and infrared spectrum) of 130 subjects in the database. Photos from various quality cameras are used to replicate real-world environments and to assess robust facial recognition algorithms, emphasizing various law enforcement and security use case scenarios. IMDB-WIKI. Since publicly accessible face picture datasets are usually limited to medium in scale, seldom reaching tens of thousands of pictures, and sometimes lack age detail, I wanted to compile a broad celebrity dataset. the list of the top 100,000 actors as listed on the IMDb website, crawled (automatically) from their profiles, including date of birth, name, gender, and all photos relevant to that user. UTKFace the dataset is a large-scale face dataset that covers a wide age range (range from 0 to 116 years old). The dataset comprises over 20,000 face photos with age, gender, and ethnicity annotations. The pictures display a wide variety of gestures, facial expressions, lighting, occlusion and clarity. Face recognition, age prediction, age progression/regression and landmark localization are only a couple of the tasks that this dataset may be used for.

Different methods are used for people counting and crowd analysis. With different methods, when it comes to a conventional method like spatial features age synthesis and estimation via face, then comes the Machine Learning technique, which can be seen to use CNN. With deeper and more sophisticated advancements of research and design, Deep Learning introduced new techniques such as age estimation. The advantages and disadvantage of methods along with references to papers for each method are presented in Table 7.

### 4.3. Action Recognition and Abnormality Detection

Detecting abnormal activities has been a great challenge and a big area that researchers have been working in to detect defects and to make the best models. One area of research is detecting abnormalities and action recognition techniques. In this section, there will be a discussion on the new trends in action recognition.

Action recognition has been researched for a long time with the increased knowledge of how humans act [75,76], using methods such as temporal templates and on space-time interest points. The techniques that were introduced first were low-level feature extraction representation and techniques. Laptev et al. [44] introduced a more advanced approach based on the approaches previously introduced. They introduced the recognition and localization of human actions.

Laptev et al. [77] completed the work of Laptev et al. [44] by also trying to train and test an algorithm in the realistic form of videos through a modern video classification approach that draws on and expands many recent concepts, including non-linear multi-channel SVMs. Dalal et al. [78] studied the grids of histograms of oriented gradient (HOG) descriptors, which did improve on the old techniques.

Lowe et al. [79] paper provided a method for removing distinctive invariant features from photographs that can be used to allow accurate correspondence between various views of an object or scene. Klaser et al. [80] presented a novel local descriptor for video sequences. Wang et al. [81] introduced trajectories of features that proved to be effective in the representation of images. They are typically extracted between frames via the KLT tracker or matching SIFT descriptors.

Wang et al. [82] cited that dense trajectories have recently been shown to be successful for action recognition and to produce outcomes on datasets. The paper enhances efficiency by taking camera motion into account to correct them using SURF descriptors and dense optical flow. Csurka et al. [83] presented a novel approach for generalized visual categorization—the issue of defining the object contents of natural images and generalizing them by differences inherent in the object class. This keypoint approach bag is based on the quantization of affine invariant image patch descriptors by a vector.

Perronnin et al. [84] introduced the Fisher kernel, which is a powerful structure within the field of pattern classification combining the qualities of generative and unequal techniques. With the introduction of new sources of Machine Learning and the increase in computational power, Deep Learning was introduced by Schmidhuber et al. [85]. In this subsection, there is a division between two CNNs, the first are the 2D CNNs and 3D CNNs.

Starting with the 2D CNNs Simonyan et al. [86] stated that the objective was to collect additional appearance information from still frames and the shift between frames. Feichtenhofer et al. [87] studied how to better take advantage of this spatio-temporal knowledge, a variety of ways to fuse the ConvNet towers both spatially and temporally.

Wang et al. [88] aimed to explore the principles for modeling successful ConvNet architectures for action recognition in videos and studying these models with samples of training. Ma et al. [89] this paper improved the preparation of deep temporal models to help understand the evolution of activity for incident prediction and early warning.

Coming to 3D CNNs introducing the literature review of different approaches and architectures that helps in action recognition. Ji et al. [90] introduced in this paper creating a novel 3D CNN model for the detection of behavior. By conducting 3D convolutions, this model derives features from both the spatial and temporal measurements, storing the motion information stored in several neighboring frames. Yet Tran et al. [91] proposed a simple but successful approach to spatiotemporal learning with deep, 3D convolution networks.

Sun et al. [92] latest attempts have been made to learn 3D CNNs for understanding human behavior in images, motivated by the popularity of convolution neural networks (CNN) for image recognition. Xie et al. [93] introduced 3D CNNs that are slightly more complex than 2D CNNs and are more vulnerable to overfitting. They pursue a systematic analysis of crucial network architecture choices by developing an accurate and efficient video classification system. Qiu et al. [94] proposed a new architecture, called Pseudo-3D Residual Net, which is a ResNet in different locations.

Varol et al. [95] studied the level of a few video frames that fail to represent behavior at their maximum temporal scale. In this study, they learn video representations using long-term temporal-convolution (LTC) neural networks. They indicate that LTC-CNN models with increased temporal extents boost the precision of the detection of behavior. Cao et al. [96] developed SlowFast video recognition networks. The model contains a Slow path, operating at a low frame rate, capturing spatial semiconductors, and a Fast path, operating at a high frame rate, capturing motion at fine time resolution.

Diba et al. [97] introduced a new spatio-temporal, deep neural network architecture named “Holistic Presence and Temporal Network” (HATNet) that builds on the convergence of 2D and 3D architectures into one by integrating intermediate representations of presence and temporal signals.

Gaidon et al. [98] proposed a paradigm based on a series of atomic action units, called “actoms”, which are semantically important and characteristic for action. The actom sequence model (ASM) describes an event as a series of histograms that are interpreted as a temporarily organized bag-of-features extension. Tian et al. [99] introduced in this paper the generalization of deform-able component models from 2D images to 3D spatiotemporal volumes to further study their video action detection effectiveness.

Shou et al. [100] produced a fixed localization of transient intervention in unregulated long images. Yeung et al. [101] introduced in their work the implementation of a complete end-to-end approach for action prediction in videos that learns to estimate the time limits of actions directly.

Escorcia et al. [102] proposed object proposals have made a major contribution to recent developments in the interpretation of objects inside images. Due to their success, they proposed Deep Action Proposals. Zhao et al. [103] proposed that an important yet challenging task is the detection of actions. In this paper, they present the Structured Segment Network, a novel framework that models each action instance’s temporal structure through a structured temporal pyramid.

Remarkable work has been done in the proposed papers that combine temporal action information with Deep Learning techniques. Table 8 has the recent most used data set when coming to action recognition. Avenue contains a small camera movement that is noticeable (in research video 2, frame 1051–1100). The training data includes a few outliers. Standard trends are rare in training results. TV Human Interaction Dataset consists of 300 video clips featuring four interactions: handshakes, high fives, embraces, and kisses, as well as clips lacking any of the interactions. The KINETICS-600 dataset contains a series of large-scale and high-quality datasets of URL links to up to 650,000 video clips that span 400/600/700 human behavior classes. Human-object encounters, such as playing instruments, as well as human–human interactions, such as holding hands and kissing, are included in the videos. There are at least 400/600/700 video samples in of action class. Each clip is 10 s long and human-annotated with a single action class.

Also, Table 9 shows the different methods used for action detection. With different methods when it comes to a conventional method like action recognition for videos by trajectory analysis. Over the years of new techniques came the Handcrafted features which present the human action classification. Then comes the Machine Learning technique which can be seen using a spatio-temporal detection technique. From more sophisticated advancements of research and design using unsupervised Deep Learning comes a new unsupervised model of action recognition. This table presents the advantages and disadvantages of the methods with references to papers on every method.

### 4.4. Fire and Smoke Detection

Detecting fire in the old days was made by detectors which detect the fire by examining the temperature threshold of the room if it exceeded a certain temperature and gives feedback of an alarm. Detectors may get false alarms and turn them on. Nowadays with the increasing amount of research the fire and smoke detection have been detected in the first stages of fire in this section, there will be a discussion on the newest techniques in the field of detection.

Tao et al. [45] introduced a new technique based on deep convolutional neural networks is proposed to enhance smoke detection precision, which can be trained end to end from raw pixel values to classifier outputs and extract features automatically from images. To introduce automated feature extraction and classification Yin et al. [118] introduced a novel deep normalization and convolution neural network (DNCNN) with 14 layers is proposed.

Yang et al. [119] combined the Gaussian Mixture Model (GMM) and the HSV color model with the video-based smoke detection deep convolution model, which helps to philter out no-smoke blocks to further reduce the rate of false detection and increase the accuracy of detection. Filonenko et al. [120] introduced the combination of a convolutional neural network (CNN) and a recurrent neural network (RNN) is recommended. The low-level features are automatically created by the CNN part, and the RNN part seeks the relationship between the features in separate frames of the same case.

Salhi et al. [121] proposed an optimized framework concept for incorporating the gas leakage and fire warning system using low-cost instruments into a standardized Machine-to-Machine (M2M) home network. Pérez-Chust et al. [122] introduced the identification of images that included pollutants or not, utilising convolutional neural networks (CNN). Then, by examining the sequences of photographs identified as having pollutants, pollutants are observed. This method of detection is based on CNN.

A 3D parallel full convolution network for wildfire smoke detection is proposed, as Li et al. [123] suggested, to segment the smoke regions in video sequences. Table 10 shows the recently used dataset in the area of Fire Detection.

Furthermore, the table displays the different methods used for people counting and crowd analysis. With different methods, conventional methods like spatial features for Fire smoke detection based on video processing using the AdaBoost technique algorithm were used. Then comes the Machine Learning technique, which can be used for the detection of early fire and smoke based on color attributes and motion detection. With deeper and more sophisticated advancements of research and design, Deep Learning introduced new techniques such as a smoke detection algorithm for an intelligent video surveillance system. Table 11 presents the advantages and disadvantages of each method, along with references for every method shown.

### 4.5. Vehicle Detection, Classification and Tracking

Detecting vehicles using old techniques has been hectic. Research in this field developed widely not only for detecting the vehicle but also for classifying it and providing the full detail of the vehicle. With the growing expansion in the field, tracking vehicles became easier due to knowing everywhere it went. In this section, there will be a complete picture of the new techniques that are widely used these days.

Annotation in manuals is fast but difficult because an expert is needed. Petrovic et al. [126] developed a model classification based on rigid structure recognition feature representation, using the distance to the Euclidean. Boyle et al. [127] tested different methods by classifying the model in 86 different classes on side-view images. HoG-RBFSVM (histogram of oriented gradients-Recognition rate–Support Vector Machinery) was best. Sochor et al. [128] uses 3D image boxes with their rasterized low-resolution shape as CNN input to classify different models. In this system, they have used You Only Look Once (YOLO) object detection architecture for localizing license plates followed by character recognition and segmentation using Convolutional Neural Networks(CNN) [129]. In [130] the moving foreground was extracted from the moving object trajectories using the GMM (Gaussian Mixture Model) technique. The vehicles were then tracked by combining an Optical Flow with a Kalman filter to estimate.

A method of counting was proposed in [131] to operate exclusively under low lighting conditions (nighttime). To detect only the vehicle’s headlights, each (grayscale) frame is the threshold by recursive image segmentation. Features like field, dimension and centroids are extracted through blob detection using Euclidean-distance.

Kim et al. [132] proposed a traffic control system that uses various digital image processing techniques and the mechanism of a convolution neural network (CNN) to identify, track, and distinguish numerous vehicles on the road in real-time. Wu et al. [133] introduced a multi-camera detection system for vehicles that greatly enhances the efficiency of identification under occlusion conditions. A novel multi-view area proposal network that locates the applicant vehicles on the ground plane comprises the main elements of the proposed process. Table 12 represents the recent frequently used data sets. GTI dataset has 3425 photographs of vehicle rears taken from multiple points of view in the archive, as well as 3900 images derived from road sequences without cars. Photos are chosen to optimize the vehicle class’s representativity, which is inherently high in uncertainty.

Additionally, Table 13 shows the comparison of methods used in Vehicle Detection, Classification, and Tracking. With different methods when it comes to conventional methods like spatial features which are Haar detection. Then comes the Machine Learning technique which can be seen using tracking by doppler radar. With deeper and more sophisticated advancements of researchers and design, Deep Learning introduced new techniques such as autonomous identification and control of occlusions and moving bodies. This table shows the methods with advantages and disadvantages with references to papers of every method.

## 5. Discussion and Future Trends

The most used nowadays COB (computer-on-board) are NVIDIA Jetson COMs, Google Coral AI, Raspberry PI, Toradex iMX series, and Inforce 6601 SoM. They give an easier use of running algorithms in a smaller size board. This enhances the integration of more than one device together so it could give a better performance for IoT devices and easy to use.

FPGA, ASIC, CPU, and GPU are used nowadays as a backbone to the hardware. The highest frequency used is x86 CPU Intel Core i7 and the least are ASIC ACCELERATORS Systolic, ASIC ACCELERATORS Eyeriss, ASIC ACCELERATORS MAERI, and FPGA Artix-7. The highest latency is ARM CPU ARMv7-A at 71.53 s and the least is FPGA Virtex-7 at 0.53 microseconds. The highest power is GPU GeForce Titan X at 230 W and the lowest power consumption is FPGA Virtex-7 at 216 mW.

For the frequently used datasets, there are lots of options when it comes to every topic. Some datasets are preferably more used by users and researchers. The most used dataset in People Counting is UCFCC50 as it has lots of people that range between 94 and 4543 with counts which make it a popular dataset in use. Then comes the Age and Gender Estimation topic where the most used nowadays dataset is IMDB-WIKI for the tremendous amount of images as it contains a wide number of images with many ranges of resolutions this gives the model a better way to train on different amount of data.

Moreover, Action Recognition and Abnormality Detection is one of the hottest topics nowadays discussed. So, there is a high demand for the datasets that are used in this topic. The most used topic is UCF101 as it gives a diversified amount of actions with large variations pose, object scale, viewpoint, cluttered background, illumination condition. Also, it has five types categories Human-Object Interaction, Body-Motion Only, Human-Human Interaction, Playing Musical Instruments, and Sports. Fire and Smoke Detection also has an enormous amount of different datasets. But, the most used nowadays is the Mivia, which is composed of 149 videos and contains smoke and fire videos, no smoke and fire videos.

Lastly, Vehicle Detection, Classification, and Tracking is a very interesting topic and booming nowadays that nearly all smart cities are trying to master. For this reason, there has been an enormous need for datasets. The most used is GTI Vehicle Image Database which has 3425 rear-angle images of vehicles on the road, as well as 3900 images of roads absent of any vehicles with also 360 × 256 pixels recorded in highways of Madrid, Brussels, and Turin.

A smart city is the city of surveillance. We see future of video surveillance is strongly tide to the evolution in smart cities, where cutting edge technologies and advanced interface between inhibitors and the system take place. Based on the review introduced above, the future trends of video surveillance could be grouped in two categories; more coexistence of sensing and computing on the side of data, and more powerful algorithms for fast and accurate services.

The leading vehicle of the advancements would be the security of the place and the safety of inhibitors. The typical current situation of a monitoring system is to preserve video recordings over a pre-defined time period to be used in the case, it is needed for security investigation related to incidents in the region under surveillance. The usual procedure in such cases is to check the recorded video streams, which is a very time-consuming and resource-demanding process. In the future, the monitoring devices will record and analyse events instantaneously. They will act as powerful guards instead of being just watchers. Even though everything could be recorded. Therefore, advances in database engines suited specially for the needs of surveillance will be needed. For example surveillance databases would be event-oriented, enhancing not just the workflow of a person seeking a particular event, but also the system’s storage space, as an entire video footage could be kept from being stored pointlessly and concentrate on saving the events.

Moreover, the communication protocols between edge surveillance devices need to be much advanced [139]. By this there could be more understanding between multiple edge devices together and intelligently speak to each other. In such process the time consumed could be decreased and the accuracy increased.

Not only protocols, but also full and smooth integration between the IoT, edge [140], fog [141], and cloud [142] computing are foreseen. This could ease the way of data extraction and transformation among sites. The implementation and operation of surveillance systems is required to promote cloud infrastructures, moving the model from standalone applications to Software-as-a-Service. This will allow the use of various video analytics and alerting mechanisms by surveillance systems as necessary and for the time period required.

New advanced powerful algorithms for video preprocessing, compression, analysis and understanding must be created. The algorithms must be able to sense and memorize important events and pop-up what could be taken as abnormal. They must be able to understand each case based on participants and context and be able to summarize results, draw conclusions and make predictions. On the other side, clearer and unambiguous policies for privacy and data security need to be formulated and implemented with very narrow tolerance.

Drones [143] are potentially the piece of technology that will contain all of that. They are becoming cheaper every day and easily manufactured. Drones can make surveillance easier as it’s easy to go places that are hard to implement cameras. They can track objects and can be adapted quickly to the situation. By this surveillance could be 24/7, every where and intelligent enough to digest data and enable action in real time.

Together with IoT based sensing cameras and devices, crowdsourcing is a important tool for surveillance. Niforatos et al. [144] proposed a crowdsourcing weather app that combines automated sensor readings from smartphones with human feedback to assess data on current and future weather events. This could benefit people in having more informative data than the ones in today’s market.

Lee et al. [145] concentrated on vehicle tracking, proposing a novel technique for tracking moving vehicles in real-time. This technique enhanced the monitoring of several vehicles at the same time and estimates of a tracker’s potential centroid region after many simulations. To improve energy efficiency and route stability, Zhang et al. [146] proposed a new routing algorithm called the Power Regulated and Stability-based Routing protocol (PCSR).

Furthermore, in [147], Al-Hader et al. attempted to solve the issue of city tree monitoring in the sense of smart cities. Since urban trees can damage cables and trigger power outages, a dynamic laser scanning device was designed to identify well-organized trees in the city.

5G is one of the topics that are being researched in for the past years. Scientists are expecting that 5G will easier life more than before with higher bandwidth and lower latency. According to Loghin et al. [148] 5G is considered to be the key enabler for smart cities, smart IoT, and effective healthcare. They looked at how 5G could aid the advancement of federated learning in this context.

## 6. Conclusions

Today’s Smart Cities’ automation policy focuses on the introduction of massive IoT (Internet of Things) networks that gather large volumes of data to obtain insights. The key goal is to make regular cities safer.

In comparison to traditional surveillance systems, which are configured for low-level operations such as tracking and recording, smart surveillance systems are expected to accommodate more applications for advanced video stream processing with large numbers of scattered edge sensors. Most progress in the area of smart surveillance has resulted in the integration of Embedded Systems and Computer Vision. Many articles have been published that provide this information.

Many articles have been published that cover a wide range of topics related to smart cities, surveillance, IoT, AI, and Edge Computing. All of the previous issues, as well as the deep organization of all areas, are expanded upon in our article. This paper fills in the gaps left by prior studies by presenting a comprehensive survey of current video security technologies and connecting them to new advances in embedded systems. Furthermore, this paper discusses the most recent datasets in use today, including extensive information on each dataset. Besides, upcoming smart city technology trends.

This paper responds to recent developments in reviews relating to computer vision (CV) technologies in smart cities by providing a thorough overview of research patterns. We go over the latest developments that might be made in the future of smart cities.

## Figures and Tables

**Table 1:** Summary of existing review papers.

Reference	Smart City	Surveillance	Internet of Things	Artificial Intelligence	Edge Computing
Dlodlo et al. [12]	🗸	×	🗸	×	×
Eigenraam et al. [18]	🗸	🗸	🗸	×	×
Roman et al. [14]	×	🗸	×	×	🗸
Bilal et al. [19]	×	×	🗸	×	🗸
Ai et al. [20]	×	×	🗸	×	🗸
Hu et al. [15]	×	🗸	×	×	🗸
Achmad et al. [21]	🗸	×	×	×	×
Yu et al. [16]	×	🗸	×	×	🗸
Gharaibeh et al. [13]	🗸	×	×	×	×
Lim et al. [22]	🗸	🗸	×	×	×
Zhaohua et al. [11]	🗸	×	🗸	🗸	×
Chen et al. [23]	🗸	×	🗸	🗸	×
Ke et al. [24]	🗸	🗸	🗸	×	×
Jameel et al. [25]	🗸	🗸	🗸	×	×
Hassan et al. [17]	×	🗸	×	×	🗸
Our review paper	🗸	🗸	🗸	🗸	🗸

**Table 2:** Different Embedded Systems used in Computer Vision.

Embedded System	Advantages	Disadvantages
NVIDIA Jetson COMs [28]	Used mainly in running TensorFlow models Used for images and video processing	More expensive than the usual COM Excessive power to work
Google Coral AI [29]	Small in Size Easily integrated with Google cloud services	Smaller version of TensorFlow models COM Steeper learning curve
Raspberry PI [30]	Excellent prototyping Easy for high production	Used for classification for small dataset COM Not the best when it comes to Computer Vision
Toradex iMX series [31]	Mainly manufacturers use it Easily programmed	Used for classification for small dataset COM Not the best when it comes to Computer Vision
Inforce 6601 SoM	Adapted for general- purpose applications	High cost

**Table 3:** FPGA, ASIC, CPU and GPU different hardware comparison with different algorithms.

Algorithm	Hardware Type	Frequency	Latency	Power
MLP [34]	FPGA spartan-6	-	800 ns	294 mW
MLP [35]	FPGA Artix-7	-	19,968 ns	123 mW
MLP [36]	FPGA zynq-7000	-	540 ns	1776 mW
MLP [36]	FPGA Artix-7	100 MHz	270 ns	240 mW
MLP [37]	FPGA Virtex-6	577 MHz	0.67 μs	372 mW
MLP [37]	FPGA Virtex-7	577 MHz	0.53 μs	216 mW
PCA(DT) [38]	Zynq SoC ZC702	-	795 ns	-
PCA(DT) [38]	Zynq SoC ZC702	-	746 ns	-
PCA(KNN) [38]	Zynq SoC ZC702	-	3073 ns	-
DNN [39]	ASIC ACCELERATORS Systolic	200 MHz	-	168 mW
DNN [39]	ASIC ACCELERATORS Eyeriss	200 MHz	-	304 mW
DNN [39]	ASIC ACCELERATORS MAERI	200 MHz	-	379 mW
DNN [40]	ASIC ACCELERATORS MERIT-z	400 MHz	-	386 mW
YOLO (Tiny) [41]	x86 CPU Intel Core i7	3.07 GHz	1.12 s	-
YOLO (Tiny) [41]	ARM CPU ARMv7-A	Upto 1 GHz	36.92 s	-
YOLO (GoogLeNet) [40]	x86 CPU Intel Core i7	3.07 GHz	13.54 s	-
YOLO (GoogLeNet) [41]	ARM CPU ARMv7-A	Upto 1 GHz	0.744 s	-
Faster RCNN (VGG16) [40]	ARM CPU ARMv7-A	Upto 1 GHz	Failed	-
YOLO (Tiny) [40]	GPU GeForce Titan X	1531 MHz	0.0037 s	178 W
SVM [41]	GeForce 840 M GPU	1.124 MHz	0.03 ms	-
SVM [41]	Kepler GPU Tegra TK1	0.852 MHz	1.23 ms	-
Markov Chain [41]	Intel i73770 CPU	1600 MHz	33.4 ns	-
Markov Chain [41]	GPUGTX690 GTX 690	1.25 GHz	Na	-
Markov Chain [41]	GPUGTX650 GTX 650	2500 MHz	Na	-
Faster RCNN (ZF) [40]	x86 CPU Intel Core i7	3.07 GHz	2.547 s	-
Faster RCNN (ZF) [40]	ARM CPU ARMv7-A	1 t GHz	71.53 s	-
Faster RCNN (ZF) [40]	FPGA Zynq (zc706)	0.2 GHz	-	-
CNN Size 2.74 GMAC [41]	Virtex6 VLX240T	150 MHz	-	-
YOLO (GoogLeNet) [40]	GPU GeForce Titan X	1531 MHz	0.010 s	230 W
Faster RCNN (ZF) [40]	GPU GeForce Titan X	1531 MHz	0.043 s	69 W
F-RCNN (VGG16) [40]	GPU GeForce Titan X	1531 MHz	0.062 s	81 W
MLP [41]	Xilinx Zynq-7000 XC7Z010T-1CLG400	-	540 ns	1.556 W
STFT and MLP [41]	FPGA Xilinx Artix-7 XC7A100T	25.237 MHz	-	0.123 W
STFT and MLP [41]	Nexys-4 Artix-7 Virtex-6 XC6VLX240T	27.889 MHz	-	3.456 W
ANN [41]	VIRTEX -E 14.5 ISE	5.332 MHz	0.6053 μs	-
SVM [41]	GPU-based Tegra TK1 System-on-Chip	875 MHz	1.23 ms	-
SVM [41]	TESLA P100 GPU	1480 MHz	0.047 ms	-
TABLA [42]	Kepler GPU Tegra TK1	852 MHz	-	5 W

**Table 4:** Frequently used datasets.

Applications	Name	Description	Type	Size and Resolution	Paper
	UCSD	- Includes a ROI and the perspective map of the scene - Contain two Ped1 and Ped2	Videos	- It is a 2000-frames video dataset from a surveillance camera of a single scene. - Ped1 contain 34 training videos and 36 testing videos while Ped2 contain 16 training videos and 12 testing videos	[57]
People Counting	UCFCC50	- Dataset contains images of extremely dense crowds - The images are collected mainly from the FLICKR	Images	- The counts of persons range between 94 and 4543 - with an average of 1280 individuals per image	[58]
	TRANCOS	- consists of 1244 images, with a total of 46,796 ve- hicles annotated - captured using the publicly available video surveillance cameras of the Direccion General de Trafico of Spain	Images	- It is a 2000-frames video dataset from a surveillance camera of a single scene	[59]

**Table 5:** Comparison between methods used in application.

Application	Method Used	Advantages	Disadvantages
People Counting	Conventional Techniques [60]	- Multiple features combined - Overcome overlap	- Count can be performed on still images only - Should be connected to a camera of kinect
	Machine Learning [61]	- Count in heavily crowded places - Detect partial human beings	- Difficult in low resolution cameras - Cannot work in dim light
	Deep Learning [62]	- Novel loss function introduced	- Time consuming when coming to training

**Table 6:** Frequently used datasets.

Applications	Name	Description	Type	Size and Resolution	Paper
Age and Gender Estimation	Age Detection of Actors	- 19,906 images in the training set - 6636 in the test set	Images	- Size: 48 MB (Compressed)	[69]
	SCFace	- Color images of faces at various angles. 4160 static images (in visible and infrared spectrum) of 130 subjects	Images	- different quality cameras mimic the real-world conditions	[70]
	UTKFace	- 21,000 frontal face images of all ages, ethnicity’s and genders	Images	- 500–600 per class	[71]
	IMDB-WIKI	- face images from 20,284 celebrities from IMDb and 62,328 from Wikipedia. List of the most popular	Images	- 523,051 Images - 27 GB for IMDB - 3 GB for WIKI - ranges of resolutions - JPEG format - 460,723 face images from IMDb and - 62,328 face images from Wikipedia	[46]

**Table 7:** Comparison between methods used in application.

Application	Method Used	Advantages	Disadvantages
Age and Gender Estimation	Conventional Techniques [72]	- Fast calculation speed - Simple to compute	- Large feature dimension - Sensitive to noise
	Machine Learning [73]	- Capture low redundancy colors - Tolerant to noise	- Works in unsupervised way - Classes are randomly chosen by the machine
	Deep Learning [74]	- the age estimation task is split into several comparative stages - very good object recognition rate	- Time consuming when coming to training - Large in dimension and could take time to train

**Table 8:** Frequently used datasets.

Applications	Name	Description	Type	Size and Resolution	Paper
	Avenue	- The videos are captured in CUHK campus avenue	Videos	- Contains 16 training and 21 testing video clips	[104]
	UMN	- Scenes are taken from inside the University of Minnesota	Videos	- 1st scene consists of 1450 frames with 320 × 240 resolution - 2nd scene consists of 4415 frames with 320 × 240 resolution - 3rd scene consists of 2145 frames with 320 × 240 resolution	[105]
	TV Human Interaction Dataset	- Videos from 20 different TV shows for prediction social actions: handshake, high five, hug, kiss and none	Videos	- 6766 video clips - 156 MB.	[106]
Action Recognition	KINETICS-600	- 500,000 videos with 600 classes	Videos	- 10 s duration - 604 GB - 392k clips	[107]
	Moments in Time Dataset	- One million labeled 3 s videos, involving people, animals, objects or natural phenomena, that capture the collected gist of a dynamic scene	Videos	- 399 classes - 1757 labeled videos per class - 3 s video duration	[108]
	SLAC	-520K untrimmed videos retrieved from YouTube	Videos	- an average length of 2.6 min- utes - 1.75 M clips, including 755K positive samples 993 K negative samples	[109]
	20-BNsometh- ingsomething Dataset V2	- a large collection of densely- labeled video clips that show humans performing pre-defined basic actions with everyday objects	Videos	- 220,847 videos - 19.4 GB - 174 classes	[110]
	Charades	- A dataset which guides our research into unstructured video activity recognition and commonsense reasoning for daily human activities. labeled video clips that show humans	Videos	- 157 action classes - 41,104 labels - 46 object classes - 27,847 textual descriptions of the videos - 76 GB - 24 fps - 480 p	[111]
	A Large-Scale Video Ben- chmark for Human Activity Underst- anding	- a wide range of complex human activities that are of interest to people in their daily living - Illustrating three scenarios in which ActivityNet can be used to compare algorithms for human activity understanding: global video classification, trimmed activity classification and activity detection	Videos	- 200 classes - 684 video hours - 10,024 training videos (15,410 instances) - 4926 validation videos (7654 instances) - 5044 testing videos (labels withheld)	[112]
	UCF101	- diversity in terms of actions and with the presence of large variations in pose, object scale, viewpoint, cluttered background, illumination condition	Videos	- action categories can be divided into five types: (1)Human-Object Interaction (2) Body-Motion Only (3) Human-Human Interaction (4) Playing Musical Instruments (5) Sports	[113]

**Table 9:** Comparison between methods used in application.

Application	Method Used	Advantages	Disadvantages
Action Detection	Trajectory analysis [114] Handcrafted features [115]	- Efficient in non-jammed scenes - Efficient for basic actions	- Can’t detect irregular shapes in jammed scenes - Not efficient for abnormal events
	Deep Learning: Supervised [116] Deep Learning: Unsupervised [117]	- Works well in understanding the behaviour - Works well in understanding the behaviour	- Both normal and abnormal events should be there - Less accurate than supervised models

**Table 10:** Frequently used datasets.

Fire Detection	Mivia	- It is composed by 149 videos	Videos	- Contains smoke and fire videos, no smoke and fire videos	[124]
	Bilkent	- Seven smoke videos and ten nonsmoke videos	Videos	- cover indoor and outdoor with different illumination, short or long distance surveillance scenes	[125]
	Cetin	- Early fire and smoke detection based on color features and motion analysis - Smoke detection sample clips	Videos		[125]

**Table 11:** Comparison between methods used in application.

Fire and Smoke detection	Conventional Techniques [72]	- Train the model to classify regions	- Low Accuracy
	Machine Learning [73]	- Classification of fire/smoke pixels using Region Segmentation	- Detection delay of about 15 s by the machine
	Deep Learning [74]	- adaptive background subtraction for movement detection	- False alarms

**Table 12:** Frequently used datasets.

Vehicle Detection /Traffic Estimation	BIT Vehicle	From the Beijing Laboratory of Intelligent Information Technology, this dataset includes 9850 vehicle images condition	Images	- six categories by vehicle type: bus, microbus, minivan, sedan, SUV, and truck	[134]
	GTI Vehicle Image Database	- 3425 rear-angle images of avehicles on the road, as well as 3900 images of roads absent of any vehicles	Images	- 360 × 256 pixels recorded in highways of Madrid, Brussels and Turin	[135]

**Table 13:** Comparison between methods used in application.

Application	Method Used	Advantages	Disadvantages
Vehicle Detection, Classification and Tracking	Conventional Techniques [136]	- Motion detection problem - Less computational time	- Background noise - Segmenting the objects from foreground nature
	Machine Learning [137]	- Provides better perf- ormance of single moving object detection based on scenario	- In multiple objects moving doesn’t work probably
	Deep Learning [138]	- Detect small objects better than multiple objects	- Restricted with the functionality of similarities

## Data Availability

All the datasets that were found in the paper have links to.
